# Supplementary material for: Genome variation and conserved regulation identify genomic regions responsible for strain specific phenotypes in rat
Source: BMC Genomics. 2017 Dec 22;18:986. doi: 10.1186/s12864-017-4351-9 (PMC5741965; doi:10.1186/s12864-017-4351-9)
Supplement: Supplementary file 1 — Supplementary figures and tables. (DOCX 724 kb) [file 12864_2017_4351_MOESM1_ESM.docx]

Additional file 1

Genome variation and conserved regulation identify genomic regions responsible for strain specific phenotypes in rat

David Martín-Gálvez^1^, Denis Dunoyer de Segonzac^1^, Man Chun John Ma^2,3,4^, Anne E Kwitek^2,3^, David Thybert^1,5^ and Paul Flicek^1^

1. European Molecular Biology Laboratory, European Bioinformatics Institute, Wellcome Genome Campus, Hinxton, Cambridge, CB10 1SD, United Kingdom

2. Department of Pharmacology, University of Iowa, Iowa City, IA, USA

3. Iowa Institute of Human Genetics, University of Iowa, Iowa City, IA, USA

4. Present address: MD Anderson Cancer Center, University of Texas, Houston, TX, USA

5. Present address: Earlham Institute, Norwich research Park, Norwich, NR4 7UH, United Kingdom

*Email addresses*

*David Martín-Gálvez: damargal@ebi.ac.uk*

*Denis Dunoyer de Segonzac: denis.desegonzac@free.fr*

*Man Chun John Ma: MMa@mdanderson.org*

*Anne E Kwitek: anne-kwitek@uiowa.edu*

*David Thybert: david.thybert@earlham.ac.uk (Corresponding author)*

*Paul Flicek: flicek@ebi.ac.uk (Corresponding author)*

#
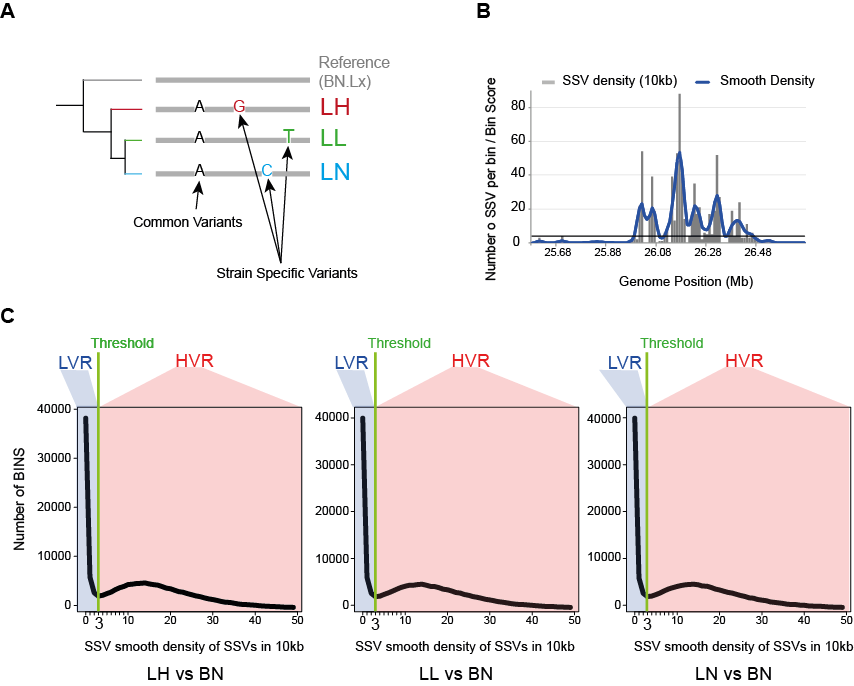
Figure S1

## Figure S1.- Procedure to identify genomic regions of interest based on the distribution of Strain-Specific Variants (SSV) across the genome.

(A) SSVs were obtained for the pairwise comparisons between the two Lyon strains susceptible to MetS (LH or LL) relative to the control Lyon rat (LHvsLN and LLvsLN) and the reference rat genome (LHvsBN and LLvsBN).

(B) Densities of SSVs were calculated in non-overlapping genome windows of 10kbp; we applied a smoothing algorithm to calculate these densities (see Methods).

(C) Distributions of the smooth densities of SSVs obtained from LHvsBN, LLvsBN and LNvsBN used to calculate the threshold to discern between High Variability Regions and Low Variability Regions. In all cases, the threshold was equal to 3.

#
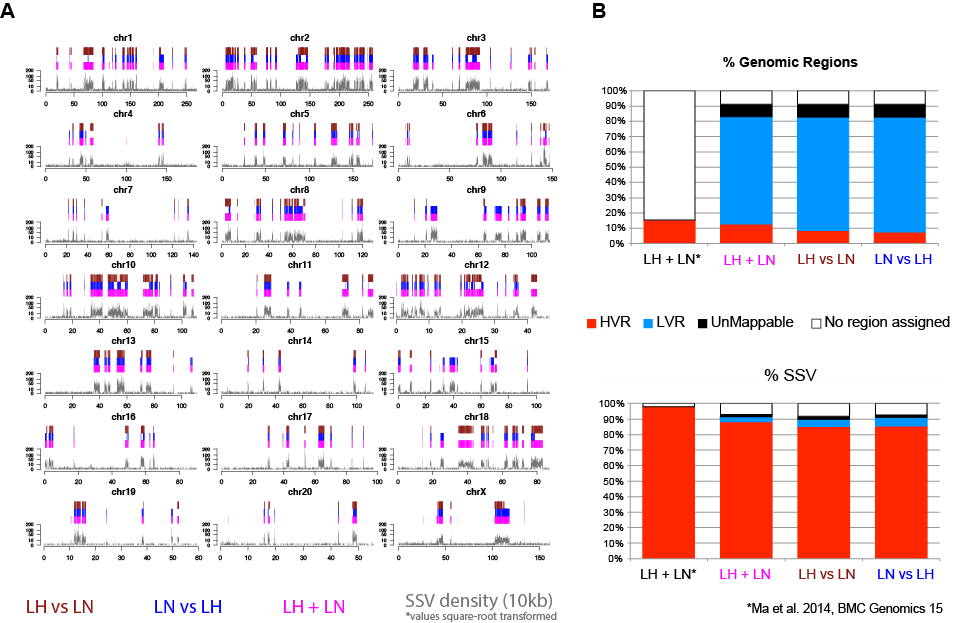
Figure S2

## Figure S2.- HVR/LVR approach in LHvsLN.

(A) Distribution of strain-specific variants (SSVs) and HVRs obtained when comparing LH and LN strains. LH+LN indicates that all genomic positions that differed between the two strains were considered as SSVs. LH vs LN indicates that SSVs were considered only if the allele that differed from the BN reference genome was in LH and was not in LN.

(B) Percentage of genome and SSVs assigned to HVRs, LVRs and UnMappables from the LH and LN comparisons. Results from Ma et al. (2014) are also shown.

#
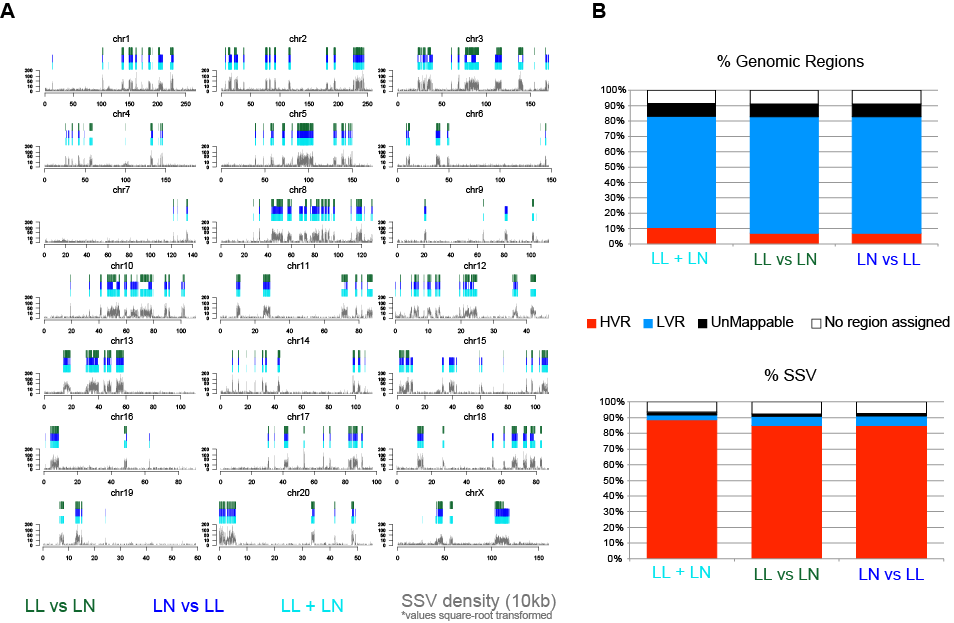
Figure S3

## Figure S3.- HVR/LVR approach in LLvsLN.

(A) Distribution of SSVs and HVRs obtained when comparing LL and LN strains. LL+LN indicates that all genomic positions that differed between the two strains were considered as SSVs. LL vs LN indicates that SSVs were considered only if the allele that differed from the BN reference genome was in LL and was not in LN.

(B) Percentage of genome and SSVs assigned to HVRs, LVRs and UnMappables from the LL and LN comparisons.

#
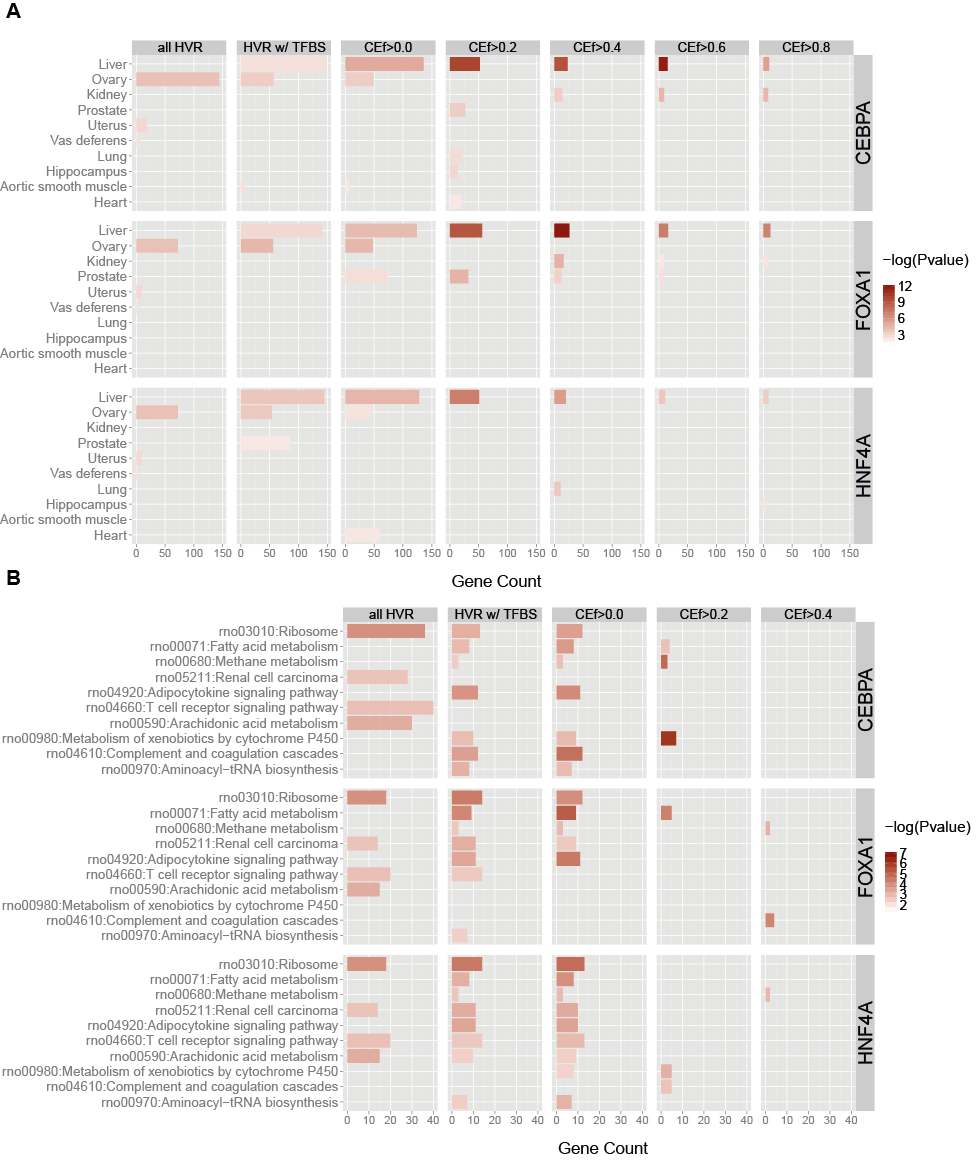
Figure S4

## Figure S4.- Gene annotation enrichment analyses performed with DAVID v6.7 in HVRs obtained for the comparison LHvsLN.

(A) Results of gene-annotation enrichment analyses performed with DAVID v6.7 with the databases UniProt Tissue and

(B) KEGG Pathway databases.

Analyses were done for each one of the subsets created from the HVRs according to the conservation of transcription factor binding sites (TFBS) between rat and mouse obtained for the three liver-specific transcription factors (CEPBA, FOXA1 and HNF4A). ‘All HVRs’ includes all HVRs, even those without any TFBS; ‘*HVRs w/ TFBS’* includes HVRs with at least one TFBS; ‘*CE_f_>0’* refers HVRs with a Conservation Enrichment (CE_f_) >0, i.e. HVRs at least one conserved TFBS between rat and mice; ‘*CEf>0.2’*, ‘*CEF>0.4’*, ‘*CEf>0.6’* and ‘*CEf>0.8’* refer to subsets of HVRs with CE_f_ greater than 0.2, 0.4, 0.6 and 0.8, respectively. Colour intensity and bar length reflect the significance (-log(p-value)) and gene count, respectively, obtained from the gene-annotation analyses for each one of the terms indicated in the y-axis. Terms associated with the databases analysed are sorted according to the accumulative significance across HVRs subsets; only the ten terms with the greatest accumulative significance are shown.

#
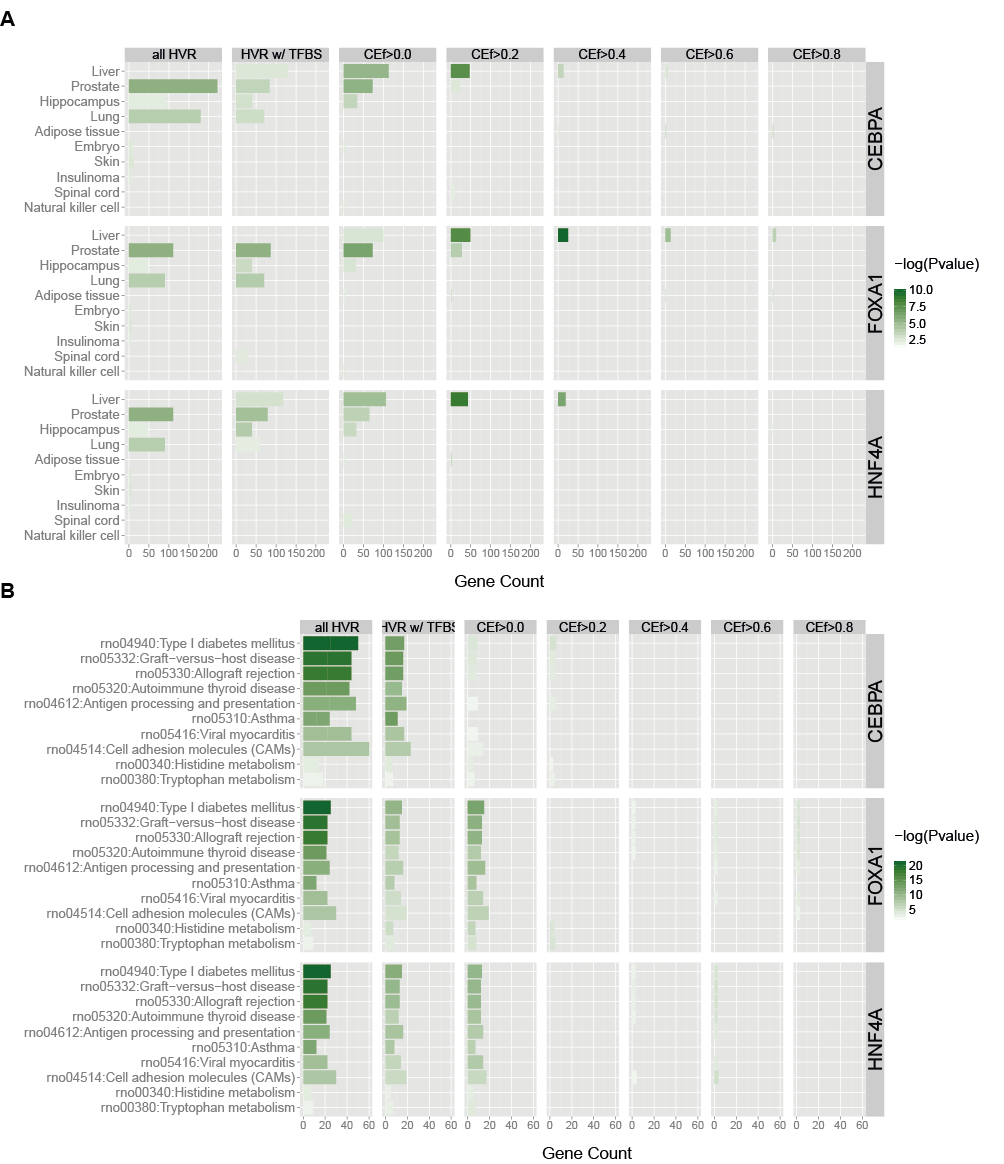
Figure S5

## Figure S5.- Gene-annotation enrichment analyses performed with DAVID v6.7 in HVRs obtained for the comparison LLvsLN.

(A) Results of gene-annotation enrichment analyses performed with DAVID v6.7 with the databases Uniprot Tissue and

(B) KEGG Pathway databases.

Analyses were done as in figure S5.

# Figure S6


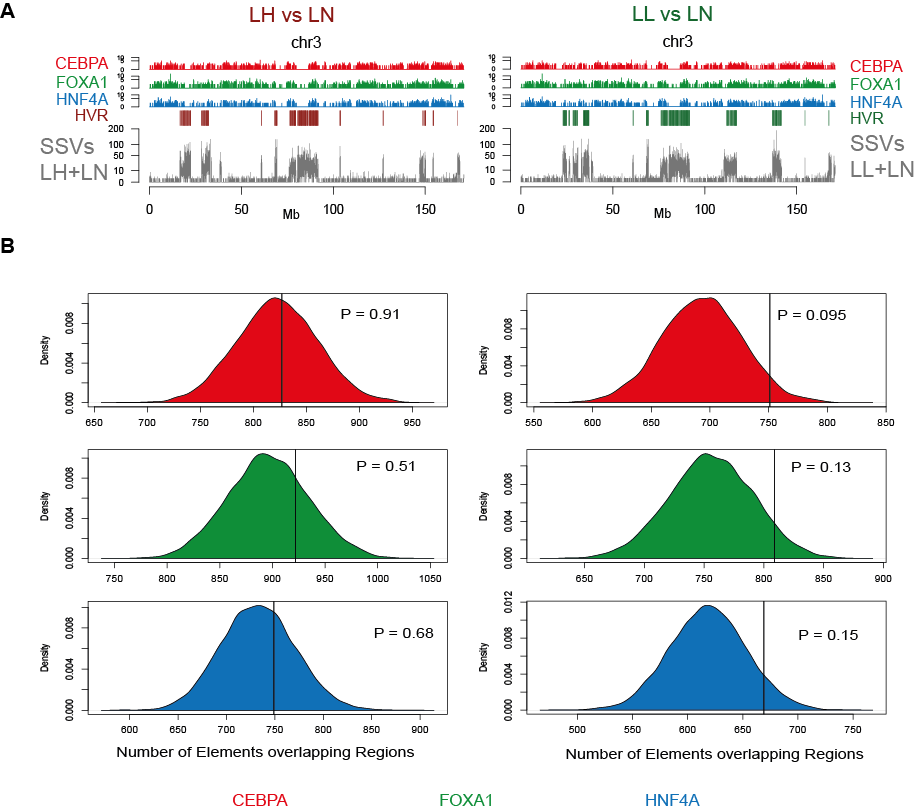


## Figure S6.- HVRs and occupancy of the occupancy for three liver-specific transcription factors.

(A) Distribution of HVRs for LHvsLN comparison across chromosome 3 and density in 10kb windows of binding sites found in BN for the three liver-specific transcription factors (CEBPA, FOXA1 and HNF4A). Density of SSVs in 10kb bins is also shown.

(B) Results from the permutations tests to analyse if the number of TFBS overlapping HVRs is significantly greater than expected by chance. Distributions are the expected values obtained from the 10,000 sets of HVRs generated by shuffling across the whole genome the coordinates of HVR clusters. The observed number of elements overlapping HVRs are indicated with a vertical line. Two-tailed p-values associated with the observed values are also shown.

# Figure S7


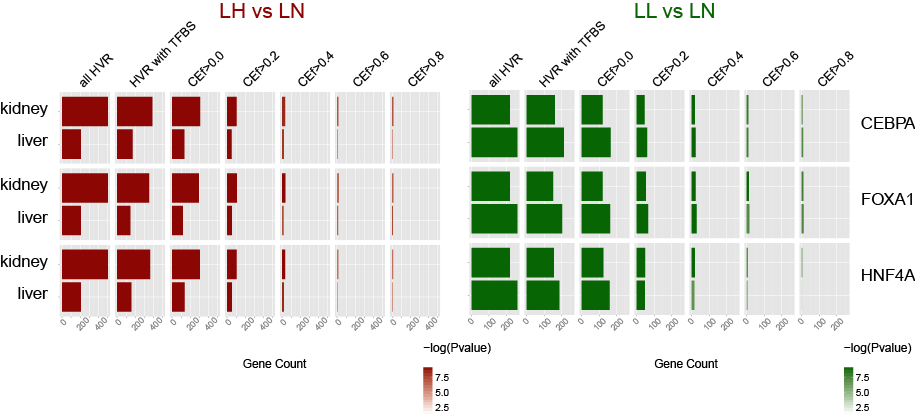


## Figure S7.- Gene expression and level of conservation level between rat and mouse of the occupancy for the three liver- specific transcription factors.

Results from the permutation analyses (10,000 permutations) to test if genes differentially expressed between LH and LN strains (A), and between LL and LN strains (B), either in liver or kidney, overlap more often than expected by chance the HVRs subsets created according to CE_f_. The colour gradient shows the significance (-log(p-value)) obtained for permutation analyses done in each one of HVR subsets. Bar size indicates the total number of liver-genes overlapping each subset of HVRs.

‘All HVRs’ includes all HVRs, even those HVRs without any transcription factor binding sites (TFBS); ‘*HVRs w/ TFBS’* includes HVRs with at least one TFBS; ‘*CEf>0’* refers HVRs with a Conservation Enrichment (*CE_f_*) >0, i.e. HVRs at least one conserved TFBS between rat and mice; ‘*CEf>0.2’*, ‘*CEf>0.4’*, ‘*CEf>0.6’* and ‘*CEf>0.8’* refer subsets of HVRs with *CE_f_* greater than 0.2, 0.4, 0.6 and 0.8, respectively

# Figure S8


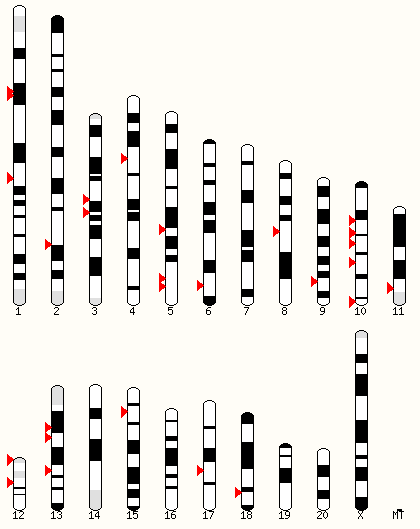


## Figure S8.- Genome localizations of rat genes having human orthologues associated with the significant enrichments for obesity

## Image modified from the Ensembl genome browser (version 69).

# Figure S9

##
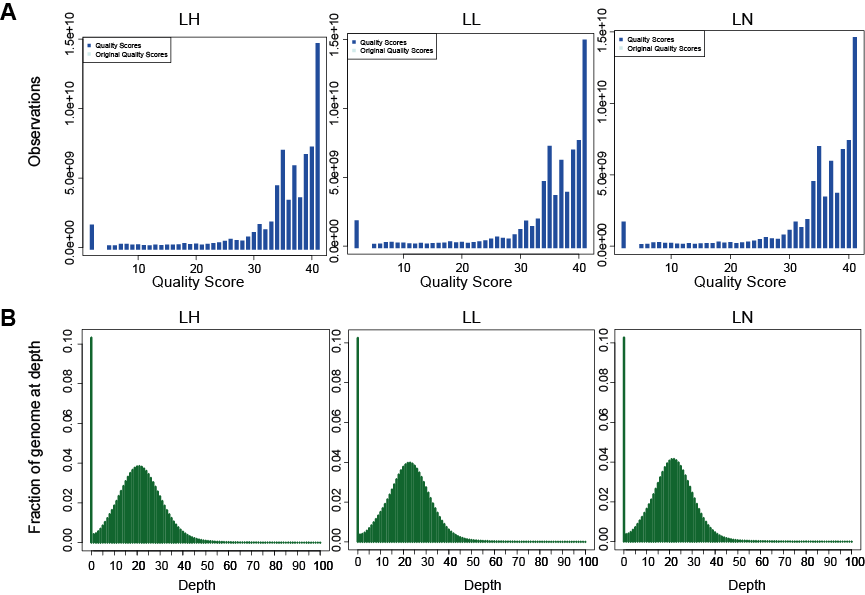
Figure S9. Accessibility of genome regions

(A) Mapping quality distributions

(B) Genome coverage of reads generated from three Lyon strains (LH, LL and LN).

We considered a genome region as non-accessible when the average of the mapping qualities is less than or equal to 30, or when the average per base coverage is greater or equal to 100.

# Figure S10


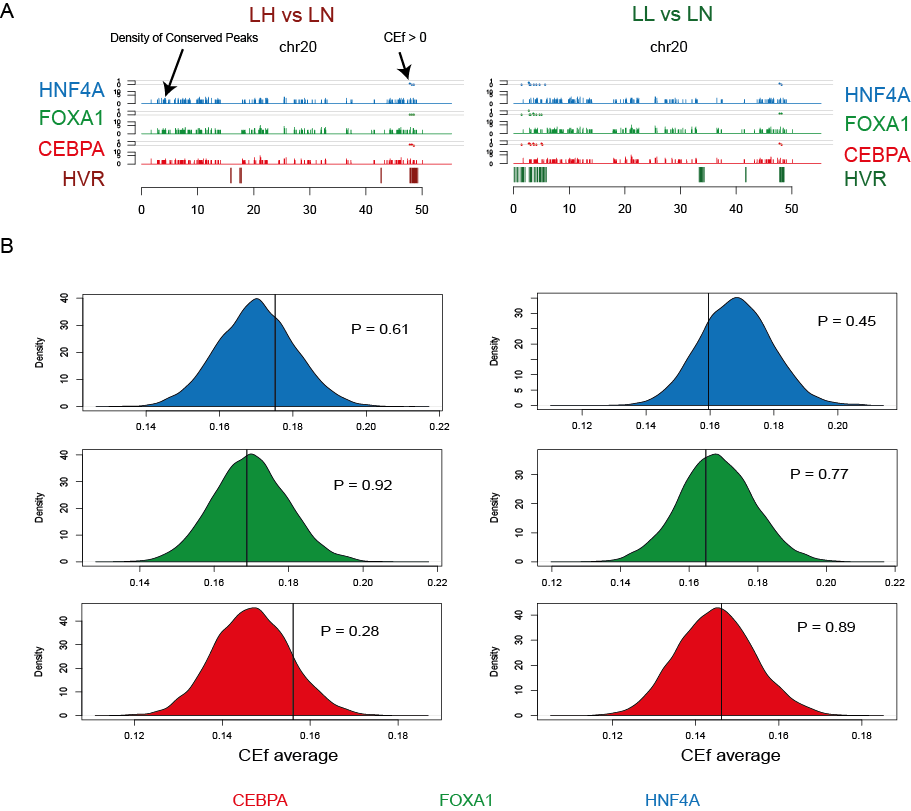


## Figure S10.- HVRs and conservation between rat and mice for the occupancy of three liver-specific transcription factors.

(A) Distribution of HVRs for LHvsLN comparison across chromosome 20 and density in 10kb windows of conserved binding sites between rat and mouse for the three liver-specific transcription factor (CEBPA, FOXA1 and HNF4A). Values of CE_f_ greater than 0 (i.e. HVRs with at least one conserved peak) are also drawn.

(B) Results from the permutations tests to analyse if average CE_f_ calculated for HVRs is significantly greater than expected by chance. Distributions of the expected values of average CE_f_ were obtained from the 10,000 sets of HVRs generated by shuffling across the whole genome the coordinates of HVR clusters. The observed values of average CE_f_ are indicated with a vertical line. Two-tailed p-values associated with the observed values are also shown.

# Table S1

| **Table S1**.- Results from analyses of liver and kidney differential gene expression between LH and LN, and between LL and LN. | | | | |
| --- | --- | --- | --- | --- |
| **Tissue** | **Strains** | **Loci not considered** | **Loci differentially expressed?** | |
|  |  |  | **Yes, total (up , down)** | **No** |
| Liver | LH vs LN | 27690 | 883 (547 , 336) | 14469 |
|  | LL vs LN | 26580 | 1847 (1038 , 809) | 15363 |
| Kidney | LH vs LN | 19654 | 2804 (1642 , 1162) | 16916 |
|  | LL vs LN | 19274 | 579 (281 , 298) | 19026 |

| Table S2.- Genes whose expression differed between LH and LN strains, and between LL and LN strains that overlapped HVRs. Expected values and two-tailed p-values were calculated from 10,000 random permutations. In all cases, the p-values obtained were the minimal value possible based on the number of permutations done. | | | | |
| --- | --- | --- | --- | --- |
| Tissue | Compared Strains | Genes overlapping at least one HVR | | two-tailed  p-value |
|  |  | Observed values | Expected values  (mean (-95% CI – +95% CI) |  |
| Liver | LH, LN | 207 | 88.28 (88.03 – 88.54) | 1×10^-4^ |
|  | LL, LN | 261 | 148.73 (148.35 – 149.1) | 1×10^-4^ |
| Kidney | LH, LN | 503 | 287.13 (286.62 – 287.64) | 1×10^-4^ |
|  | LL, LN | 218 | 46.83 (46.62 – 47.03) | 1×10^-4^ |

# Table S2

# Table S3

| **Table S3.-** Terms used and count of GWAS variants obtained from the NHGRI-EBI GWAS catalogue (1119 GWA SNPs, accessed from Ensembl v87) and that were mapped to rat orthologous regions (474 SNPs, assembly Rno6.0) | |
| --- | --- |
| Bilirubin levels in extreme obesity | 1 |
| Hepatic lipid content in extreme obesity | 1 |
| Metabolic syndrome | 25 |
| Metabolic syndrome (bivariate traits) | 4 |
| Metabolic traits | 17 |
| Obesity | 45 |
| Obesity (early onset extreme) | 10 |
| Obesity (extreme) | 7 |
| Obesity and osteoporosis | 1 |
| Obesity in adult survivors of childhood cancer exposed to cranial radiation | 1 |
| Obesity-related traits | 344 |
| Thiazide-induced adverse metabolic effects in hypertensive patients | 21 |
| **Total** | **474*^§^** |
| *five of these variants were assigned to two different terms: rs3101336, rs1558902 and rs1421085 to Obesity / Obesity (early onset extreme)), rs673548 to Metabolic syndrome / Metabolic traits, and rs2206277 to Obesity / Metabolic syndrome (bivariate traits).  **^§^** 56 of these variants were lost during the conversion to the RGSC3.4 assembly | |

# Table S4

| **Table S4.-** Enrichment of rat orthologous positions of human GWAS variants associated with metabolic traits. OV is the number variants overlapping HVR subsets. P-values were calculated from permutation tests. | | | | | | | | | | | |
| --- | --- | --- | --- | --- | --- | --- | --- | --- | --- | --- | --- |
| **Strain** | **TF** | **all HVR** | | **HVR w/ TFBS** | | **CE_f_>0.0** | | **CE_f_>0.2** | | **CE_f_>0.4** | |
|  |  | **OV** | **p-value** | **OV** | **p-value** | **OV** | **p-value** | **OV** | **p-value** | **OV** | **p-value** |
| LH | CEBPA | 45 | 0.1376 | 29 | 0.0135 | 19 | 0.0844 | 7 | 0.0258 | 1 | 1 |
|  | FOXA1 | 45 | 0.1331 | 30 | 0.0061 | 21 | 0.027 | 9 | 0.0138 | 4 | 0.0221 |
|  | HNF4A | 45 | 0.1355 | 29 | 0.0033 | 21 | 0.0174 | 8 | 0.0178 | 2 | 0.1671 |
| LL | CEBPA | 34 | 0.4451 | 23 | 0.0624 | 16 | 0.0706 | 5 | 0.083 | 1 | 1 |
|  | FOXA1 | 34 | 0.4383 | 24 | 0.0344 | 19 | 0.0142 | 11 | 0.0031 | 7 | 0.002 |
|  | HNF4A | 34 | 0.4448 | 22 | 0.0256 | 18 | 0.0135 | 8 | 0.0096 | 5 | 0.0025 |

# Table S5

| **Table S5.-** Enrichment of rat orthologous positions of human GWAS variants associated with metabolic traits. OV is the number variants overlapping HVR subsets. P-values were calculated from permutation tests. | | | |
| --- | --- | --- | --- |
| **Strain** | **HVR subset** | **OV** | **p-value** |
| LH | 1TF | 26 | 0.0254 |
|  | 2TF | 21 | 0.02 |
|  | 3TF | 14 | 0.0796 |
| LL | 1TF | 21 | 0.0396 |
|  | 2TF | 18 | 0.0161 |
|  | 3TF | 14 | 0.0225 |

**Table S6**

| **Table S6.-**  Genes one-to-one assigned to promoters with strain-specific variants (SSVs) in the HVRs that were selected from using our approach in LHvsLN and whose human orthologues were associated with obesity. Also shown is assignment to liver from UP_Tissue database and differential expression status in liver or kidney with respect to the control Lyon rat (LN). | | | | | | | | | | |
| --- | --- | --- | --- | --- | --- | --- | --- | --- | --- | --- |
| **Ensembl ID Rat** | **Coord (RGSC3.4, chr:start-end)** | **strains** | **Liver-gene** | **dif-liver-gene** | **dif-kidney-gene** | **SSVs** | **GWAS** | **Ensembl ID Human** | **Hgnc name** | **PMID^§^** |
| ENSRNOG00000001457 | 12:23185552-23198655 | LHvsLN_LLvsLN | no | no_no | no_no | 1 | 0 | ENSG00000049541 | RFC2 | 1 |
| ENSRNOG00000001720 | 11:72603851-72606212 | LHvsLN_LLvsLN | no | no_no | no_no | 6 | 0 | ENSG00000114315 | HES1 | 2 |
| ENSRNOG00000002598 | 10:72081121-72186997 | LHvsLN_LLvsLN | yes | no_no | no_no | 13 | 0 | ENSG00000108753 | HNF1B | 2 |
| ENSRNOG00000002778 | 10:72701735-72794495 | LHvsLN_LLvsLN | no | no_no | no_no | 1 | 1 | ENSG00000108270 | AATF | 1 |
| ENSRNOG00000002823 | 10:35344672-35384319 | LHvsLN | no | yes | no | 8 | 0 | ENSG00000050748 | MAPK9 | 1 |
| ENSRNOG00000002896 | 13:76824440-76835531 | LHvsLN | no | no | yes | 4 | 0 | ENSG00000117592 | PRDX6 | 1 |
| ENSRNOG00000002946 | 10:107958636-107959313 | LHvsLN | yes | yes | yes | 26 | 0 | ENSG00000184557 | SOCS3 | 21 |
| ENSRNOG00000003226 | 10:45975683-46090595 | LHvsLN_LLvsLN | no | no_no | no_no | 2 | 0 | ENSG00000133030 | MPRIP | 1 |
| ENSRNOG00000003463 | 10:46462216-46483620 | LHvsLN_LLvsLN | no | yes_no | no_no | 32 | 0 | ENSG00000072310 | SREBF1 | 16 |
| ENSRNOG00000004143 | 13:47358625-47379562 | LHvsLN_LLvsLN | no | no_no | no_no | 2 | 0 | ENSG00000159346 | ADIPOR1 | 25 |
| ENSRNOG00000004827 | 6:129936041-129988323 | LHvsLN | no | no | no | 6 | 0 | ENSG00000090060 | PAPOLA | 1 |
| ENSRNOG00000007060 | 5:105718496-105732817 | LHvsLN | no | no | no | 6 | 0 | ENSG00000147872 | PLIN2 | 1 |
| ENSRNOG00000007387 | 10:55859851-55868747 | LHvsLN | yes | yes | yes | 1 | 0 | ENSG00000179094 | PER1 | 2 |
| ENSRNOG00000007437 | 4:56407560-56418694 | LHvsLN_LLvsLN | no | no_no | no_no | 1 | 0 | ENSG00000128604 | IRF5 | 1 |
| ENSRNOG00000007478 | 3:76800939-76830893 | LHvsLN_LLvsLN | no | no_no | no_no | 1 | 0 | ENSG00000121671 | CRY2 | 2 |
| ENSRNOG00000008364 | 3:88654131-88686212 | LHvsLN_LLvsLN | yes | no_no | no_no | 58 | 0 | ENSG00000121691 | CAT | 2 |
| ENSRNOG00000010645 | 15:23327071-23339006 | LHvsLN | no | yes | yes | 6 | 0 | ENSG00000131981 | LGALS3 | 3 |
| ENSRNOG00000011039 | 15:23120339-23139794 | LHvsLN | yes | yes | no | 8 | 0 | ENSG00000131979 | GCH1 | 1 |
| ENSRNOG00000011927 | 5:149487938-149518917 | LHvsLN | no | no | no | 1 | 0 | ENSG00000162512 | SDC3 | 2 |
| ENSRNOG00000012404 | 1:154647341-154651663 | LHvsLN_LLvsLN | yes | yes_yes | yes_no | 24 | 0 | ENSG00000151365 | THRSP | 3 |
| ENSRNOG00000013954 | 5:156501744-156558749 | LHvsLN | yes | no | no | 1 | 0 | ENSG00000162551 | ALPL | 1 |
| ENSRNOG00000014900 | 17:62770633-62837668 | LHvsLN | no | no | no | 3 | 0 | ENSG00000095794 | CREM | 1 |
| ENSRNOG00000016066 | 17:62654080-62658885 | LHvsLN | no | no | yes | 2 | 0 | ENSG00000095739 | BAMBI | 1 |
| ENSRNOG00000016770 | 1:77244169-77252808 | LHvsLN | no | no | no | 2 | 0 | ENSG00000160014 | CALM3 | 1 |
| ENSRNOG00000018359 | 18:72294803-72323350 | LHvsLN | no | yes | no | 40 | 0 | ENSG00000101665 | SMAD7 | 1 |
| ENSRNOG00000020392 | 2:204395554-204439388 | LHvsLN | no | no | no | 10 | 0 | ENSG00000116266 | STXBP3 | 1 |
| ENSRNOG00000020652 | 1:80894705-80911019 | LHvsLN | yes | no | no | 2 | 0 | ENSG00000105329 | TGFB1 | 13 |
| ENSRNOG00000021586 | 13:38182153-38400278 | LHvsLN_LLvsLN | no | no_no | no_no | 1 | 0 | ENSG00000183840 | GPR39 | 1 |
| ENSRNOG00000023548 | 9:92509650-92567345 | LHvsLN | no | no | yes | 1 | 0 | ENSG00000162804 | SNED1 | 1 |
| ENSRNOG00000023856 | 9:92412128-92422075 | LHvsLN | yes | no | no | 22 | 0 | ENSG00000172482 | AGXT | 3 |
| ENSRNOG00000026171 | 8:63153456-63189904 | LHvsLN | no | no | no | 2 | 0 | ENSG00000140463 | BBS4 | 5 |
| ENSRNOG00000029986 | 12:2934967-3087691 | LHvsLN | no | no | no | 7 | 0 | ENSG00000171105 | INSR | 32 |
| § Sum of PubMed IDs reporting GDAs in DisGenet database (v4.0, http://www.disgenet.org) between a given gene and obesity. | | | | | | | | | | |

| **Table S7.-** Functional Enrichment Analyses done by GeneMANIA for the 32 human orthologues associated with the significant enrichment for obesity in LH strain. | | |  |  |
| --- | --- | --- | --- | --- |
| **GO id** | **Description** | **q-value** | **Occurrences in Sample** | **Occurrences in Genome** |
| GO:0007623 | circadian rhythm | 3.04E-09 | 8 | 42 |
| GO:0048511 | rhythmic process | 1.02E-07 | 8 | 69 |
| GO:0032922 | circadian regulation of gene expression | 8.96E-07 | 5 | 13 |
| GO:0044212 | transcription regulatory region DNA binding | 1.39E-03 | 8 | 267 |
| GO:0000975 | regulatory region DNA binding | 1.39E-03 | 8 | 268 |
| GO:0001067 | regulatory region nucleic acid binding | 1.39E-03 | 8 | 268 |
| GO:0030512 | negative regulation of transforming growth factor beta receptor signalling pathway | 1.39E-03 | 5 | 57 |
| GO:0090288 | negative regulation of cellular response to growth factor stimulus | 3.72E-03 | 5 | 74 |
| GO:0090101 | negative regulation of transmembrane receptor protein serine/threonine kinase signalling pathway | 3.87E-03 | 5 | 78 |
| GO:0017015 | regulation of transforming growth factor beta receptor signalling pathway | 3.87E-03 | 5 | 77 |
| GO:0001047 | core promoter binding | 1.58E-02 | 4 | 50 |
| GO:0005160 | transforming growth factor beta receptor binding | 1.58E-02 | 3 | 16 |
| GO:0051259 | protein oligomerization | 1.84E-02 | 6 | 194 |
| GO:0051260 | protein homooligomerization | 1.95E-02 | 5 | 116 |
| GO:0090287 | regulation of cellular response to growth factor stimulus | 1.98E-02 | 5 | 118 |
| GO:0009416 | response to light stimulus | 2.40E-02 | 6 | 211 |
| GO:0090092 | regulation of transmembrane receptor protein serine/threonine kinase signalling pathway | 3.00E-02 | 5 | 132 |
| GO:2000736 | regulation of stem cell differentiation | 3.88E-02 | 4 | 70 |
| GO:0042752 | regulation of circadian rhythm | 4.02E-02 | 3 | 25 |
| GO:0007179 | transforming growth factor beta receptor signalling pathway | 4.70E-02 | 5 | 150 |
| Colours that GO IDs are associated with genes belong to the same module, which were obtained from the network analyses based on protein-protein interaction (see Figure 6) | | | | |

**Table S8**

| **Table S8** **.**- Overlap between candidate genes reported by Ma et al. 2014 and Wang et al. 2015 and genes with strain-specific variation associated with the HVR 3TF set by using the conservation between mouse and rat in occupancy of the three liver-specific transcription factors. | | | | | | | | | |
| --- | --- | --- | --- | --- | --- | --- | --- | --- | --- |
| **Ensembl ID** | **NS-SSVs** | **Promoters** | **Study** | **Coordinates (RGSC-3.4)** | **Ensembl ID Human** | **Hgnc name** | **PMIDs Insulin Resistance^§^** | **PMIDs Dyslipidaemias^§^** | **PMIDs Obesity^§^** |
| *ENSRNOG00000000142* |  |  | Ma et al. 2014 | 17:90572391-90982073 | ENSG00000120594 | PLXDC2 |  |  |  |
| *ENSRNOG00000000279* |  |  | Wang et al. 2015 | 20:47818499-47857741 | ENSG00000130347 | RTN4IP1 |  |  |  |
| *ENSRNOG00000002618* |  |  | Wang et al. 2015 | 13:66223323-66235533 | ENSG00000116679 | IVNS1ABP |  |  | 1 |
| *ENSRNOG00000002724* |  |  | Wang et al. 2015 | 10:47890410-47925430 | ENSG00000141127 | PRPSAP2 |  |  |  |
| *ENSRNOG00000002823* |  |  | Wang et al. 2015 | 10:35344672-35384319 | ENSG00000050748 | MAPK9 |  |  | 1 |
| *ENSRNOG00000002994* |  |  | Wang et al. 2015 | 11:64154287-64170282 | ENSG00000121577 | POPDC2 |  |  |  |
| *ENSRNOG00000003070* |  |  | Wang et al. 2015 | 10:48629286-48689381 | ENSG00000108474 | PIGL |  |  |  |
| *ENSRNOG00000007379* |  |  | Wang et al. 2015 | 6:41875020-41882235 | ENSG00000134330 | IAH1 |  |  |  |
| *ENSRNOG00000007845* |  |  | Wang et al. 2015 | 10:76030806-76036988 | ENSG00000213246 | SUPT4H1 |  |  |  |
| *ENSRNOG00000010630* |  |  | Wang et al. 2015 | 1:149711289-149763868 | ENSG00000137509 | PRCP |  |  | 2 |
| *ENSRNOG00000012126* |  |  | Ma et al. 2014 | 17:53402078-53436081 | ENSG00000010270 | STARD3NL |  |  |  |
| *ENSRNOG00000012418* |  |  | Ma et al. 2014 | 17:53496423-53528130 |  |  |  |  |  |
| *ENSRNOG00000012490* |  |  | Ma et al. 2014 | 17:53558804-53802936 | ENSG00000078053 | AMPH |  |  |  |
| *ENSRNOG00000013358* |  |  | Wang et al. 2015 | 1:154973798-154983989 | ENSG00000178301 | AQP11 |  |  |  |
| *ENSRNOG00000014834* | yes |  | Ma et al. 2014 | 17:63041415-63104046 | ENSG00000120616 | EPC1 |  |  |  |
| *ENSRNOG00000014900* |  |  | Ma et al. 2014 | 17:62770633-62837668 | ENSG00000095794 | CREM |  |  | 1 |
| *ENSRNOG00000015292* |  |  | Ma et al. 2014 | 17:62701289-62741344 | ENSG00000108094 | CUL2 |  |  |  |
| *ENSRNOG00000015437* |  |  | Ma et al. 2014 | 17:29701923-29708044 |  |  |  |  |  |
| *ENSRNOG00000016066* |  |  | Ma et al. 2014 | 17:62654080-62658885 | ENSG00000095739 | BAMBI |  |  | 1 |
| *ENSRNOG00000016109* | yes |  | Wang et al. 2015 | 10:56745822-56757435 | ENSG00000215041 | NEURL4 |  |  |  |
| *ENSRNOG00000016307* |  |  | Ma et al. 2014 | 17:43119152-43126570 |  |  |  |  |  |
| *ENSRNOG00000016436* |  |  | Ma et al. 2014 | 17:43276214-43284152 |  |  |  |  |  |
| *ENSRNOG00000017187* |  |  | Wang et al. 2015 | 2:205909919-205915148 | ENSG00000198890 | PRMT6 |  |  |  |
| *ENSRNOG00000018698* |  |  | Ma et al. 2014 | 17:64531772-64587027 | ENSG00000095787 | WAC |  |  |  |
| *ENSRNOG00000018760* |  |  | Ma et al. 2014 | 17:63992387-64282554 | ENSG00000150054 | MPP7 |  |  |  |
| *ENSRNOG00000018905* |  |  | Ma et al. 2014 | 17:63931393-63955410 | ENSG00000169126 | ARMC4 |  |  |  |
| *ENSRNOG00000018938* |  |  | Ma et al. 2014 | 17:63631426-63710099 | ENSG00000150051 | MKX |  |  |  |
| *ENSRNOG00000018972* |  |  | Ma et al. 2014 | 17:63497924-63529227 | ENSG00000099246 | RAB18 |  |  | 1 |
| *ENSRNOG00000019659* |  |  | Wang et al. 2015 | 10:60178512-60199209 | ENSG00000108381 | ASPA |  |  | 4 |
| *ENSRNOG00000019994* |  |  | Wang et al. 2015 | 1:79893158-79910727 | ENSG00000176531 | PHLDB3 |  |  |  |
| *ENSRNOG00000021216* |  |  | Wang et al. 2015 | 2:191437355-191446244 | ENSG00000131779 | PEX11B |  |  |  |
| *ENSRNOG00000022378* | yes |  | Ma et al. 2014 | 17:65681793-65702382 |  |  |  |  |  |
| *ENSRNOG00000023778* |  |  | Ma et al. 2014 | 17:29767388-29872873 | ENSG00000111846 | GCNT2 |  |  |  |
| *ENSRNOG00000023799* |  |  | Ma et al. 2014 | 17:29710407-29721477 | ENSG00000111845 | PAK1IP1 |  |  |  |
| *ENSRNOG00000025630* |  |  | Ma et al. 2014 | 17:42228845-42229431 |  |  |  |  |  |
| *ENSRNOG00000027453* | yes |  | Ma et al. 2014 | 17:62684244-62686747 |  |  |  |  |  |
| *ENSRNOG00000027571* |  |  | Ma et al. 2014 | 17:53441303-53484317 |  |  |  |  |  |
| *ENSRNOG00000031981* |  |  | Ma et al. 2014 | 17:83837499-83861361 |  |  |  |  |  |
| *ENSRNOG00000033865* |  |  | Wang et al. 2015 | 10:39407540-39417112 |  |  |  |  |  |
| *ENSRNOG00000038737* |  |  | Ma et al. 2014 | 17:53773036-53773733 |  |  |  |  |  |
| *ENSRNOG00000039075* |  |  | Ma et al. 2014 | 17:42984939-42991275 |  |  |  |  |  |
| *ENSRNOG00000039379* |  |  | Ma et al. 2014 / Wang et al. 2015 | 17:29733271-29746769 |  |  |  |  |  |
| *ENSRNOG00000043379* |  |  | Ma et al. 2014 | 17:30267398-30267637 | ENSG00000137434 | C6orf52 |  |  |  |
| ***NS-SSVs*** indicates that the gene contains non-synonymous strain-specific variation overlapping the selected HVRs  ***Promoter*** indicates if promoters linked to the genes held strain-specific variation overlapping the selected HVRs  § ***PMIDs insulin resistance***, ***PMIDs dyslipidaemias*** and ***PMIDs obesity*** show the sum of PubMed IDs reporting evidence in DisGenet database (v4.0, http://www.disgenet.org) for association between a given gene and insulin resistance, dyslipidaemias and obesity, respectively. | | | | | | | | | |

# Table S9

| **Table S9-** Size (in Mb) of the sets of HVRs selected according to Conservation Enrichment for each TF. | | | | | | | | |
| --- | --- | --- | --- | --- | --- | --- | --- | --- |
| **Strains** | **TF** | **All HVRs** | **HVRs**  **w/ TFBS** | **CE_f_>0.0** | **CE_f_>0.2** | **CE_f_>0.4** | **CE_f_>0.6** | **CE_f_>0.8** |
| LHvsLN | CEBPA | 232.36 | 114.36 | 81.65 | 16.48 | 4.65 | 1.65 | 1.27 |
|  | FOXA1 | 232.36 | 111.17 | 79.89 | 20.87 | 6.34 | 3.00 | 1.56 |
|  | HNF4A | 232.36 | 101.23 | 75.83 | 18.19 | 4.93 | 2.20 | 1.20 |
|  |  |  |  |  |  |  |  |  |
| LLvsLN | CEBPA | 193.06 | 97.83 | 63.89 | 14.72 | 4.23 | 1.23 | 0.81 |
|  | FOXA1 | 193.06 | 96.26 | 65.26 | 18.54 | 6.13 | 2.98 | 1.27 |
|  | HNF4A | 193.06 | 85.42 | 59.68 | 14.58 | 4.38 | 1.62 | 0.68 |

# Table S10

| **Table S10.-** Strains Specific Variants (SSVs) in the sets of HVRs selected according to Conservation Enrichment for each TF. | | | | | | | | |
| --- | --- | --- | --- | --- | --- | --- | --- | --- |
| **Strains** | **Tf** | **All HVRs** | **HVRs**  **w/ TFBS** | **CE_f_>0.0** | **CE_f_>0.2** | **CE_f_>0.4** | **CE_f_>0.6** | **CE_f_>0.8** |
| LHvsLN | CEBPA | 351,460 | 164,069 | 116,056 | 21,292 | 5,847 | 2,479 | 1,954 |
|  | FOXA1 | 351,460 | 162,132 | 115,858 | 27,520 | 7,917 | 3,767 | 2,147 |
|  | HNF4A | 351,460 | 146,196 | 108,836 | 23,708 | 6,085 | 2,634 | 1,473 |
|  |  |  |  |  |  |  |  |  |
| LLvsLN | CEBPA | 276,054 | 134,070 | 87,065 | 18,644 | 5,159 | 1,561 | 1,144 |
|  | FOXA1 | 276,054 | 134,128 | 90,446 | 23,878 | 7,310 | 3,705 | 1,657 |
|  | HNF4A | 276,054 | 118,644 | 82,031 | 18,894 | 5,383 | 1,926 | 854 |

#

# Table S11

| **Table S11.-** Size (in Mb) and Strains Specific Variants (SSVs) of the sets of HVRs selected according to number of liver-specific TFs with conserved peaks. | | | |
| --- | --- | --- | --- |
| **Strain** | **HVR subset** | **Size in Mb** | **Num. SSVs** |
| LHvsLN | 1TF | 105.24 | 150147 |
|  | 2TF | 76.67 | 110469 |
|  | 3TF | 55.46 | 80134 |
|  |  |  |  |
| LLvsLN | 1TF | 82.89 | 112172 |
|  | 2TF | 60.7 | 83866 |
|  | 3TF | 45.24 | 63504 |
